# Cognitive appraisal, emotional responses, and emotion regulation in firefighters' occupational stress—A pilot study

**DOI:** 10.3389/fpubh.2026.1809101

**Published:** 2026-05-26

**Authors:** Pauline Georgees Poless, Jarle Eid, Mathilde V. Bjercke Prate, Elida Margareth Stenseng, Anita Lill Hansen

**Affiliations:** 1 Department of Psychosocial Science, University of Bergen, Bergen, Norway; 2 Centre for Research and Education in Forensic Psychiatry, Haukeland University Hospital, Bergen, Norway

**Keywords:** cognitive-emotional processes, cognitive reappraisal, first responders, Socratic questioning, suppression

## Abstract

**Introduction:**

Firefighting is a highly demanding profession, involving frequent exposure to stress and life-threatening situations. This pilot study investigates firefighters' cognitive and emotional responses underlying problem-solving and emotion regulation during stressful operations. It explores how and to what extent firefighters experience their thoughts and emotions, focusing on subjective experiences of valence and physiological arousal, and whether they perceive their thoughts and emotions as helpful or hindrances. Additionally, we examine habitual emotion regulation strategies, accounting for age and work experience.

**Method:**

Seventy-eight firefighters participated in structured interviews based on Cognitive Behavioural Therapy techniques and completed the Emotion Regulation Questionnaire (ERQ).

**Results:**

Fire accidents were reported as the most stressful operations (35%), with cognitive appraisal oriented to stress-threat (31%). Participants experienced mixed emotions (27%) rather than a single dominant emotion and consistently prioritised rescuing individuals as a problem-solving tendency (49%). Despite high subjective experience of bodily changes and physiological arousal, thoughts and emotions were generally perceived as positive and helpful. Cognitive reappraisal was the most frequently used emotion regulation strategy both during stressful operations (23%) and as a habitual tendency, while suppression was less common and negatively correlated with age. Age explained approximately 8% of the variance in suppression.

**Discussion:**

By using a Cognitive Behavioural Therapy (CBT) framework, this pilot study demonstrates new insight into firefighters' underlying cognitive and emotional processes during critical operations. Significant strengths and limitations, as well as suggestions for further research are discussed.

## 1 Introduction

Firefighters face exceptional physical and psychological demands in their line of work. The operational environment requires rapid cognitive appraisal, precise task execution, and flexible emotional regulation. Consequently, firefighter training and development must foster an adaptive response to high-stakes demands such as fires, traffic accidents, or rescue missions (1). This preparation includes training to handle physical hazards such as extreme heat, thermal injuries, falls, and exposure to toxic combustion by-products [e.g., (2, 3)].

Research has identified a range of occupational and environmental stressors affecting firefighters, including mechanical (3, 4), physical (5), chemical hazards (6), and leadership-related challenges (7). The cumulative impact of these work-related stressors has been consistently associated with adverse physical and psychological outcomes (8). Given their frequent exposure to acute stress, it is essential to explore how firefighters appraise, feel, act, and manage their emotions in response to acute operational stressors. However, the cognitive appraisals that underlie emotional responses, problem-solving, and emotion regulation in these contexts remain insufficiently understood (9). This pilot study contributes to close this gap by providing evidence-based information in support of screening, resilience training, and targeted debriefing interventions (10).

The *Transactional Theory of Stress and Coping* (11) provides a comprehensive framework for understanding individual differences in stress responses. According to Lazarus and Folkman (11), stress does not stem directly from external events but rather from how individuals appraise and interpret these events through a dynamic cognitive process. Consequently, factors such as age and operational experience can significantly shape how firefighters perceive and respond to a fire and rescue situation. For instance, Goh et al. (12) found that younger men with extensive firefighting experience were more susceptible to psychological distress than older colleagues with similar levels of experience. The authors suggest that age and experience may moderate the impact of operational exposure on psychological outcomes.

Lazarus and Folkman (11) describe cognitive appraisal as comprising two interrelated components: *primary* and *secondary appraisal*. In primary appraisal, individuals evaluate the significance of an event, determining whether it is irrelevant, positive, or *stressful*. Stressful events are further categorised as harm/loss, threat, or challenge, each of which may evoke predominantly negative emotions due to perceived or actual danger. In the secondary appraisal, individuals assess their available coping resources and strategies, including perceived control, coping capacity, and prior experience. Emotions play a central role in this appraisal process, influencing both the interpretation of the situation and the selection of coping responses (11).

Behavioural tendencies that follow primary and secondary appraisals play a crucial role in how individuals manage stressful situations (11). For fire and rescue personnel, coping strategies usually fall into two categories. Problem-focused coping involves directly tackling the source of stress when possible, while emotion-focused coping aims to manage emotional responses when the situation is beyond their control (13). Although resolving operational challenges is often a priority, it is not always possible. Consequently, psychological strain may persist even when missions are deemed successful (14). Given this, research on firefighting should emphasise the importance of long-term approaches to managing emotional overload. Gaining insight such requires revisiting the individual's appraisal of the triggering situation, the emotions it elicits, and the coping mechanisms employed. Emotion regulation functions as a higher-order component of the stress response, allowing individuals to adjust their emotional states based on subjective cognitive appraisals (15). Rather than operating in isolation, emotion regulation is embedded within the broader cognitive-affective processing system. Among the various strategies individuals use to manage intense emotional experiences, two have received particular attention: *cognitive reappraisal* and *suppression* (15, 16). Cognitive reappraisal involves reinterpreting a stressful situation to reduce its emotional impact. It is widely considered an adaptive strategy, associated with enhanced psychological wellbeing and improved social functioning. In contrast, suppression, which entails emotional expression, is generally viewed as less adaptive, often associated with increased stress and psychological discomfort (17, 18).

Individuals' beliefs about the *helpfulness* of emotions, along with their subjective experiences of *valence* (positive or negative emotions) and physiological *arousal* (perceived intensity of emotional activation), play a critical role in emotion regulation. Viewing emotions as helpful tends to promote adaptive strategies, such as cognitive reappraisal, whereas perceiving emotions as disruptive or undesirable is associated with maladaptive strategies, like emotional suppression (19). When emotions are seen as negative and undesirable, individuals are more likely to rely on suppression or avoidance. In contrast, perceiving emotions as valuable and informative facilitates more flexible and constructive regulation (20). Effective emotion regulation also requires an adequate level of arousal and engagement, as both positive and negative regulation efforts can enhance subjective experience and expressive behaviour (21, 22).

Given the profound impact that emotion regulation strategies have on mental health, performance and operational effectiveness, it is essential to examine how firefighters apply these strategies in practise. However, the existing literature remains limited in this area, often focusing instead on related constructs such as emotional intelligence (57), social support, and coping mechanisms (23–25). The purpose of this pilot study is to gain a broader understanding of cognitive-emotional processes underlying problem-solving and emotion-regulation strategies related to occupational stress among firefighters. The study is divided into two analytical aims:

*Cognitive-emotional processes in firefighters' occupational stress*: The first analytical aim is to gain new insights into firefighters' typical cognitive appraisals and emotional responses during stressful operations, as well as the resulting outcomes, such as problem-solving and emotion regulation. Additionally, we aimed to investigate how, and to what extent, firefighters experience their thoughts and emotions, focusing on valence [e.g., (20)], subjective experiences of physiological arousal [e.g., (21, 22)], and whether they perceive their thoughts and emotions as helpful or hindrance [e.g., (19)].

*Emotion regulation as a habitual tendency*: The second analytical aim examines emotion regulation as a stable, habitual tendency drawing on the process model of emotion regulation by Gross and John (15). This section explores how firefighters typically manage their emotions in various situations, independent of specific operational stressful contexts. We account for age and work experience in our analyses, as these factors have been shown to correlate with psychological distress among firefighters (12).

## 2 Materials and methods

### 2.1 Participants

The research involved 78 firefighters, including 44 fire constables and 30 operational leaders from a REDACTED covering approximately 290,000 citizens. The sample consisted of five females and 73 males, aged 26 to 61 years (M = 42.01, SD = 9.06). Selection criteria emphasised practical experience in emergency operations, including firefighting, life-saving missions, and first responder procedures. Participants had 2–40 years of work experience in the firefighting field.

### 2.2 Research design

The pilot study used a cross-sectional design and employed both structured interviews and self-report questionnaires to address the research aims.

#### 2.2.1 Structured interview

A structured interview was employed to collect in-depth insights into firefighters' cognitive appraisals, emotional responses, problem-solving and emotional regulation in stressful and critical operations. The interview framework was based on the ABC model developed by Ellis (56), which illustrates the interactions between Activating Events (A), Beliefs (B), and Consequences (C) (see Table 1). To access these factors, the Socratic questioning technique was employed (26, 55). This approach encouraged participants to actively re-engage with their experiences rather than merely recounting them, thereby facilitating a deeper understanding of the underlying cognitive and emotional processes. The interview guide was developed by a certified Cognitive Behavioural Therapy (CBT) therapist and is grounded in CBT principles. Interviews were conducted by the first author of this article and graduate students in psychology. All of whom were trained and supervised by a certified CBT therapist to ensure consistency and adherence to cognitive-behavioural principles.

**Table 1:** Interview guide based on cognitive behavioural therapy principles.

Activating event (A)	Belief (B)	Consequences (C)			
Situation	Cognitive appraisal	Emotions	Subjective physiological reaction (Arousal)	Problem-solving	Emotion regulation in operations
**Question 1:** Do you have an example of an event, situation, or dilemma that you found particularly challenging or difficult (i.e., a task that was hard to resolve and triggered a high degree of stress or discomfort)? **Question 2:** On a scale from 0 to 8, how severe/extreme/dangerous/uncomfortable was the event?	**Question 3:** If you now try to think back to that event, do you remember what you thought or how you assessed the situation? **Question 4:** On a scale from 0 to 8, how negative/positive (e.g., optimistic, confident in your ability to cope) was the thought? **Question 5:** If we take a step back and evaluate the helpfulness of the thought, how useful/helpful was it in your work on a scale from 0 to 8?	**Question 6:** Did this situation evoke any emotions in you? (Alternatively, simply ask which emotions arose during this event.) **Question 7:** On a scale from 0 to 8, how uncomfortable (negative) or comfortable (positive) was the emotion? **Question 8:** In hindsight, and on a scale from 0 to 8, how helpful was the emotion? In other words, did you experience the emotion as an obstacle, or did it help you navigate the situation/perform your work?	**Question 9:** On a scale from 0 to 8, how activated did you feel (how intense was the emotion)?	**Question 10:** What was the outcome of this situation? Do you remember what you decided to do and how you solved the problem?	**Question 11:** Do you remember how you handled or dealt with the thoughts and emotions that arose during the situation? ***Helping questions:*** – So, did you do anything to change your thoughts and feelings in the situation? – How did you get through the situation? For example, was there something specific you said to yourself to get through the incident? Do you remember if this affected your thoughts/feelings?

Questions 1, 3, 6, 10, and 11 gave qualitative answers, while questions 2, 4, 5, 6, 7, 8, and 9 gave quantitative answers.

Interview questions required a combination of qualitative and quantitative responses (see Table 1). Qualitative questions explored firefighters' previously experienced operational situations (Question 1) to access concrete underlying cognitive appraisals (i.e., the content of their thoughts; Question 3), specific emotions elicited during the operation (Question 6), the problem-solving strategies used (Question 10), and how they regulated their emotions throughout the operation (Question 11). In addition, participants were asked to rate their responses to each exploratory question (i.e., Questions 2, 4, 5, 6, 7, 8, and 9) on Likert-type scales ranging from 0 to 8 (e.g., 0 = very negative/low, 4 = neutral, 8 = very positive/high). This approach allowed us to measure how, and to what extent, participants experienced their thoughts and emotions in terms of valence (positive or negative) and perceived usefulness (helpful or hindrance). The CBT-question technique also makes it possible to explore participants' subjective experiences of bodily and physiological changes, such as increased arousal (e.g., increased heart rate) during the operation (Question 9). The complete interview guide is presented in Table 1.

#### 2.2.2 Self-rapport questionnaire

To assess habitual emotion regulation strategies, participants completed the Norwegian version of the Emotion Regulation Scale [ERS (15)]. The ERS is a 10-item self-report questionnaire (scored 1 = strongly disagree to 10 = strongly agree) that assesses tendencies to suppress emotions (six items) and to reappraise emotions (four items). Suppression items include statements such as “*I control my emotions by not expressing them*.” Reappraisal items include statements such as “*I control my emotions by changing the way I think about the situation I am in*.” In our study, the scale demonstrated acceptable internal consistency for reappraisal (Cronbach's α = 0.78) and suppression items (Cronbach's α = 0.72).

### 2.3 Procedures

The participants were recruited with assistance from a senior staff officer at five fire stations in Norwegian municipalities. Individual interviews were conducted over 6 months in 2023–24. These interviews took place at the respective fire stations where the firefighters were stationed. Interviews were scheduled after duty hours in a private room at each station, lasted between 25 and 30 min, and were transcribed verbatim. To ensure participant privacy, no audio or video recordings were made. Following each interview, participants completed the Emotion Regulation Scale [ERS (15)] in paper-and-pencil format, which took approximately 10 min.

### 2.4 Ethical considerations

The study protocol was approved by the Norwegian Agency for Shared Services in Education and Research (Ref. code: 194439). The participants provided their verbal and/or written consent and received information about their right to withdraw from the study at any time without consequences.

### 2.5 Data analysis

For analytical aim 1, we employed template analysis to explore the most frequently occurring qualitative responses collected through the interviews. Template analysis is a flexible form of thematic analysis that combines a structured coding approach with a sensitivity to nuances in the data (27). This method involves developing a predefined coding *template*, which is iteratively refined in response to the empirical material.

Thus, template analysis was used to identify typical cognitive appraisal and thoughts, emotional responses, problem-solving and emotion regulation during stressful operations. The coding of *cognitive appraisal* and *problem-solving* was guided by the Transactional Theory of Stress and Coping (11). To assess *emotional responses* during stressful operations, we applied Ekman's (28) theory of basic emotions, which includes fear, anger, happiness, sadness, disgust, and surprise, as a theoretical framework. Regarding emotion regulation in stressful operations, we anticipated identifying tendencies towards cognitive reappraisal and suppression, consistent with Gross and John's (15) theoretical framework.

Based on established evidence of the robustness of ANOVA to non-normal and Likert-scale data, a Repeated-measures ANOVA was used for the present analyses [e.g., (29–31)]. All firefighters were treated as a single group, and responses to the following interview questions were treated as dependent variables: *Question 4:* On a scale from 0 to 8, how negative/positive was the thought? (valence of thought), *Question 5*: If we take a step back and evaluate the helpfulness of the thought, how useful/helpful was it in your work on a scale from 0 to 8? (thought helpfulness), *Question 7:* On a scale from 0 to 8, how uncomfortable (negative) or comfortable (positive) was the emotion? (emotional valence), *Question 8*: In hindsight, and on a scale from 0 to 8, how helpful was the emotion? In other words, did you experience the emotion as an obstacle, or did it help you navigate the situation/perform your work? (emotion helpfulness) and *Question 9*: On a scale from 0 to 8, how activated/aroused did you feel (how intense was the emotion)? (subjective experience of bodily changes and physiological arousal). Relationships among the dependent variables were also explored using Pearson's bivariate correlation. Statistical analyses were conducted using IBM SPSS Statistics, version 29.0.2.0 (32).

For analytical aim 2, we examined cognitive reappraisal and suppression as relatively stable emotion regulation strategies. Descriptive statistics were calculated to illustrate the distributions of these strategies, along with the variables age and work experience. A paired-samples *t*-test was performed to evaluate the mean differences between cognitive reappraisal and suppression. Correlation analyses were conducted to investigate the associations among cognitive reappraisal, suppression, age and work experiences. Linear regression analyses are included as follow-up analyses in cases where significant correlations were found.

## 3 Results

### 3.1 Analytical aim 1

#### 3.1.1 Cognitive-emotional processes in firefighters' occupational stress

The template analysis of the qualitative interview responses indicated that the two most prevalent categories of *stressful operations* were fire-related accidents (35%) and traffic-related accidents (26%). In terms of *cognitive appraisal*, stress-threat emerged as the most dominant form of primary appraisal (31%), followed by a problem-solving focus (23%), representing secondary appraisal. Regarding *emotional responses*, the most frequently reported experience was the presence of multiple or mixed emotions during stressful incidents (27%), rather than a single discrete emotion. This was followed by fear and stress, each reported by 12% of the sample. For *problem-solving*, the most reported strategy was rescuing people in danger (49%), followed by adhering to established rescue procedures (18%). In terms of *emotion regulation during stressful operations*, the sample showed a clear tendency towards cognitive reappraisal (23%). Notably, acceptance also emerged as a frequently used emotion regulation strategy, reported by 19% of the sample. Table 2 presents two of the most frequently reported themes along with illustrative citations.

**Table 2:** Examples of the most frequently occurring themes and illustrative citations.

Activating event Stressful situation	Belief Cognitive appraisal	Consequences		
		Emotions response	Problem-solving	Emotion regulation in operations
**Fire accidents** “*House fire. Father and four children inside. I knew the father personally”* **Traffic Accidents** “*Traffic accident. Five people in the car. Parents died. Children rescued*.”	**Primary appraisal stress: threat** “*Thought about our safety. If we don't get out now, we might die*.” **Secondary Appraisal Problem-solving focus** “*Work mode. What should be prioritised? What needs to be done. Ask others what needs to be done*.”	**Combined emotions** “*Stress. Surprised. A bit happy. Disappointed, could have been more efficient*.” **Fear** “*Fear. Fear of death*.” **Stress** “*Stressed. Activated. Alert*.”	**Rescuing people in danger** “*Freeing a child. Securing the car against fire*.” **Followed the rescue procedure** “*Procedural work. Handle the injuries. Secure the car*.”	**Reappraisal** “*Mentally prepared in advance. I was prepared for it not to go well. Calm enough to carry out the task*.” **Acceptance** “*First experience – just going with the flow and accepting*.”

Illustrative citations from different participants.

#### 3.1.2 Subjective experiences of thoughts and emotions

To examine how and to what extent participants experienced their thoughts and emotions a Repeated Measures ANOVA was performed. The results revealed a significant main effect of condition across the five variables: *Thought valence* (Question 4), *thought helpfulness* (Question 5), *emotion valence* (Question 7), *emotion helpfulness* (Question 8), and *emotion arousal* (subjective experience of bodily changes and physiological arousal; Question 9), *F*_(4, 304)_ = 28.17, *p* < 0.001, partial η^2^ = 0.27. The assumption of sphericity was met, W = 0.86, χ^2^_(9)_ = 11.53, *p* = 0.24, and multivariate tests confirmed this finding [Wilks' Λ = 0.43, *F*_(4, 73)_ = 24.73, *p* < 0.001].

**Figure 1:**
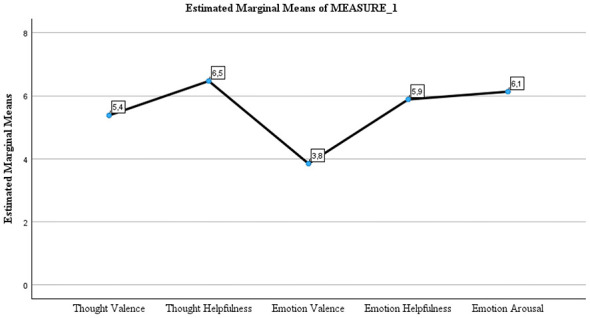
A visual summary of the estimated means of subjective experiences of thoughts and emotions in fire and rescue operations. Thought Valence (0 = Very negative, 4, neutral, 8 = Very positive), Thought Helpfulness (0 = a significant hindrance, 4 = neutral, 8 = very helpful); Emotion Valence (0 = Very negative, 4, neutral, 8 = Very positive); Emotion Helpfulness (0 = a significant hindrance, 4 = neutral, 8 = very helpful); Emotion Arousal (0 = very low, 4 = neutral, 8 = very high).

Analysis of the estimated means indicated that *emotion valence* was rated lowest (M = 3.84), suggesting that participants generally experienced negative emotions during stressful operations, with a slight tendency towards neutrality. In contrast, *thought helpfulness* was rated highest (M = 6.47), indicating that the firefighters perceived their thoughts as highly useful in managing the operational stressful situations. Subjective experience of bodily changes and physiological arousal (*emotion arousal*, Question 9) was also rated relatively high (M = 6.13), reflecting a strong sense of emotional activation during stressful operations (see Table 3 for an overview of the means and standard deviations for all dependent variables). Figure 1 presents a visual summary of these findings.

**Table 3:** Descriptive statistics and correlations between variables.

Variables	Mean	SD	Min	Max	95% CI	1	2	3	4
1. Reappraisal	29.8	4.6	20	42	[28.80, 30.97]	–			
2. Suppression	13.9	4.3	4	24	[13.05, 14.99]	−0.028 [−0.269, 0.224]	–		
3. Age	42.0	9.0	26	61	[40.03, 44.40]	0.28 [−0.214, 0.258]	−0.283^*^ [−0.514, −0.042]	–	
4. Work experience	14.8	9.9	2	40	[12.54, 17.15]	−0.017 [−0.247, 0.215]	−0.20 [−0.440, 0.052]	0.856^**^ [0.758, 0.927]	–

^*^Correlation is significant at the 0.05 level. ^**^Correlation is significant at the 0.01 level. Values in square brackets indicate the 95% confidence for each correlation. The bold values indicate statistically significant findings.

#### 3.1.3 Correlational relationships between thoughts and emotions

Looking at the correlational relationships between variables measuring how participants experienced their thought and emotions, the results showed a positive correlation between *thought valence* and *emotion valence* (*r* = 0.25, *p* < 0.05). Further, *emotion helpfulness* correlated positively with both *thought helpfulness* (*r* = 0.35, *p* < 0.01) and *emotion valence* (*r* = 0.25, *p* < 0.05). Additionally, *emotion arousal* was positively correlated with *emotion helpfulness* (*r* = 0.26, *p* < 0.05) and *thought helpfulness* (*r* = 0.25, *p* < 0.05), indicating that thoughts and emotions are deemed more helpful, there is also an increase in subjective experience of bodily changes and physiological arousal. Overall, these findings suggest that more positive and beneficial thoughts are linked to more positive and helpful emotional experiences during stressful operations among firefighters (see also Table 4).

**Table 4:** Descriptive statistics and correlations between variables.

Variables	Mean	SD	Min	Max	95% CI	1	2	3	4	5
1. Thought valence	5.4	2.1	0	8	[4.9, 5.8]	–				
2. Thought helpfulness	6.4	1.8	0	8	[6.0, 6.8]	0.210 [−0.047, 0.448]	–			
3. Emotion valence	3.8	1.8	0	8	[3.4, 4.2]	0.254^*^ [−0.005, 0.476]	0.179 [−0.039, 0.401]	–		
4. Emotion helpfulness	5.9	1.6	1	8	[5.5, 6.2]	0.195 [−0.027, 0.415]	0.346^**^ [0.095, 0.599]	0.246^*^ [−0.003, 0.462]	–	
5. Emotion arousal	6.1	1.8	0	8	[1.5, 2.1]	0.140 [−0.134, 0.408]	0.250^*^ [0.018, 0.483]	−0.036 [−0.283, 0.212]	0.262^*^ [0.076, 0.449]	–

^*^Correlation is significant at the 0.05 level. ^**^Correlation is significant at the 0.01 level. Thought Valence (Question 4), Thought Helpfulness (Question 5), Emotion Valence (Question 7), Emotion Helpfulness (Question 8), Emotion Arousal (Question 9). Values in square brackets indicate the 95% confidence for each correlation. The bold values indicate statistically significant findings.

### 3.2 Analytical aim 2

#### 3.2.1 Habitual emotion regulation strategies

Participants reported a mean score of 29.8 for cognitive reappraisal (M = 29.8, SD = 4.6), with a range of 20–42. The mean suppression score was 13.97 (M = 13.9, SD = 4.3), with a range of 4–24. A paired-samples *t*-test was conducted to examine mean differences between cognitive reappraisal and suppression. The analysis revealed a statistically significant difference, *t*_(73)_ = 21.24, *p* < 0.001, with firefighters reporting significantly higher reappraisal scores than suppression scores. The mean difference was 15.8 (SD = 6.43), with a 95% confidence interval of [14.39, 17.37]. The effect size was large (d = 2.47), indicating a substantial difference between the two emotion regulation strategies.

#### 3.2.2 Emotion regulation strategies, age, and work experiences

To examine whether emotion regulation strategies, reappraisal and suppression, are related to age and work experience, correlational analyses were conducted. A negative correlation was found between age and suppression (*r* = −0.28, *p* < 0.05), indicating that older participants reported lower levels of suppression. No significant correlation was observed between age and reappraisal (*r* = 0.28, ns), nor between reappraisal and suppression (*r* = −0.03, ns). Work experience did not correlate with any of the emotion regulation strategies (see Table 3).

#### 3.2.3 Follow-up analysis: age as a predictor of suppression

A simple linear regression was conducted to examine whether age predicts the variance in suppression, given the observed negative correlation between the two variables. The model was significant, *F*_(1, 72)_ = 6.29, *p* = 0.014, explaining 8% of the variance in suppression (*R*^2^ = 0.08, adjusted *R*^2^ = 0.07). Age was negatively associated with suppression (β = −0.28, *t* = −2.51, *p* = 0.014), indicating that older participants reported lower levels of suppression. The unstandardized coefficient (B = −0.134, SE = 0.05) indicates that suppression decreases by 0.13 units per additional year of age. The results are shown in Table 5.

**Table 5:** Linear regression age predicting suppression.

Variables	B	SE B	β	*t*	*p*	95% CI
Constant	19.62	2.30	–	8.52	< 0.001	15.02, 24.20
Age	−0.134	0.05	−0.283	−2.51	0.014	−0.240, −0.027

*R* = 0.28, *R*^2^ = 0.08, *F*_(1, 72)_ = 6.29, *p* = 0.014.

## 4 Discussion

Employing a CBT framework, the current pilot study offers new insights into how firefighters evaluate stressful operations, respond emotionally, solve problems, and regulate their emotions during such events. Moreover, the CBT approach provides a clearer understanding of the interplay between firefighters' thoughts and emotions. Furthermore, analysis of habitual emotion regulation, specifically cognitive reappraisal and suppression, reveals a stronger tendency towards cognitive reappraisal than suppression. Notably, age demonstrates a negative association with suppression, suggesting that older firefighters are less inclined to employ this regulatory strategy.

### 4.1 Cognitive-emotional processes in firefighters' occupational stress

The first aim of this study was to gain new insights into firefighters' typical cognitive appraisals and emotional responses during critical, stressful fire and rescue operations, as well as the outcomes that follow, including problem-solving and emotion regulation. The results from template analyses indicated that the most stressful operations predominantly involved *fire* and *traffic accidents*. The majority evaluated such incidents primarily as *stress–threat* scenarios, while the second most common appraisal was a *problem-solving focus*, emphasising the actions required to manage the situation effectively. This finding aligns well with Lazarus and Folkman's (11) Transactional Theory of Stress and Coping by illustrating the differences between primary and secondary appraisal processes. From Table 2, the *stress–threat* (e.g., “*Thought about our safety. If we do not get out now, we might die*.”) reflects an anticipation of potential harm or loss that has not yet occurred but is perceived as imminent. In contrast, Table 2 also illustrates the *problem-solving focus* (e.g., “*Work mode. What should be prioritised? What needs to be done? Ask others what needs to be done*”) that aligns with secondary appraisal, emphasising coping options and perceived control. The interaction between secondary and primary appraisals of what is at risk plays a crucial role in determining both the intensity of stress and the quality of the emotional response (11). When *stress–threat* predominates, particularly in contexts of low perceived control, emotional strain tends to increase. Empirical evidence indicates that *stress–threat* appraisals are strongly associated with adverse mental health outcomes. In other words, those who tend to perceive stress as a *threat* are less able to manage it than those who see it as a challenge (33). Nevertheless, various individual differences and personality traits matter for stress-related appraisals and emotional processes. Some traits are more closely linked to primary appraisals, while others are connected to secondary appraisals. For example, neuroticism is strongly associated with threat-related stress appraisals, whereas extraversion tends to be linked to challenge-related stress appraisals [e.g., (34)].

Conversely, a task-oriented focus may promote more adaptive coping and facilitate effective action even under high situational demands. Firefighters are a highly trained, selectively recruited group, specifically prepared to manage and respond to such high-risk scenarios. Consequently, they are more frequently exposed to these situations. Research conducted by Baumann et al. (35) indicates that firefighters who have practised risk scenarios are more likely to experience less anxiety and cognitive difficulties during operations and are better equipped to handle such situations effectively. The fact that firefighters report both threat-related stress and a problem-solving coping approach provides valuable insight into the emotional and cognitive strain they may experience in stressful operations. At the same time, it highlights their ability to maintain a task-oriented focus and engage in problem-solving even under demanding, high-pressure conditions.

Regarding the emotional responses that arose in stressful situations, the firefighters reported experiencing emotional complexity in terms of a range of different emotions (e.g., “*Stress. Surprised. A bit happy. Disappointed*”). This tendency for firefighters to experience multiple emotions might indicate a complex cognitive and appraisal process in which different factors, such as perceived threat, responsibility, time pressure, and control, trigger distinct emotional responses. Ashkanasy and Dorris (36) suggest that professionals often experience multiple emotions simultaneously due to layered social, cognitive, and contextual factors. Emotional complexity is normal and can be a sign of deep engagement and care. Previous research indicates that experiencing multiple emotions simultaneously or within a specific timeframe is often viewed as indicative of greater emotional complexity and more adaptive functioning (37). *Fear* and *stress* were the next most frequently reported emotions among participants. Experiences of those emotions align with previous research from high-risk contexts, where the safety of oneself and others is at stake [e.g., (38)]. Those emotions, particularly stress, can be functional in that they mobilise performance under pressure [e.g., (39, 40)], but they can also be dysfunctional, in limiting flexibility and increasing cognitive load [e.g., (41, 42)]. In other words, it is normal for firefighters in demanding situations to experience negative emotions like fear and stress, and in doing so, they mobilise for action. However, it can be dysfunctional if it impairs adaptive functioning in operational situations.

The fact that firefighters identify a *problem-solving focus* as their second-most reported cognitive appraisal is evident in how they approach and resolve challenging and stressful operations. Regarding problem-solving, participants showed a strong focus on *saving lives* (e.g., “*Freeing a child. Securing the car against fire”*) and closely *following established procedures* (e.g., “*Procedural work. Handle the injuries. Secure the car*.”). This pattern aligns with previous research showing that firefighters tend to exhibit pronounced problem-solving tendencies (24, 43). In operational contexts, firefighters tend to rely on problem-focused coping strategies. They gather information, formulate action plans, and implement concrete solutions, rather than engaging in emotion-focused avoidance, particularly during the most intense phases of an incident (13). Together, these findings indicate that problem-solving appraisal is closely linked to observable behaviours in the field. Firefighters tend to prioritise rescues and adhere to standard operating procedures to manage complex, high-pressure situations effectively.

The way firefighters handle and resolve high-stress situations is critical, not only for their own safety but also for the safety of others. Equally vital is their ability to cope with the cognitive and emotional challenges that emerge during these intense incidents, as these factors have direct repercussions on mental health, psychological wellbeing, and overall job performance (44, 45). Effective emotion regulation plays a key role in this context, allowing firefighters to manage their cognitive and emotional responses while maintaining focus and adapting their performance under pressure. The firefighters participating in this study reported that *cognitive reappraisal* was the most frequently used emotion regulation strategy in stressful operations, followed closely by *acceptance*. Cognitive reappraisal involves altering one's interpretation of a situation to change its emotional impact and is associated with reduced symptoms of various psychological disorders (46). In contrast, acceptance involves recognising and embracing emotional responses without attempting to change them, which can facilitate emotional processing and minimise avoidance behaviours (47). The preference for cognitive reappraisal and acceptance over suppression likely reflects their adaptive advantages. For firefighters, who are routinely exposed to high-stress and potentially traumatic situations, these strategies support psychological resilience and effective functioning. In contrast, suppression, which involves inhibiting the outward expression of emotions, has been associated with poorer mental health outcomes (46). The fact that they tend to employ adaptive emotion regulation, which is vital in managing negative emotions, shows that firefighters generally regulate their emotions adaptively.

We also sought to investigate how, and to what extent, firefighters experience their thoughts and emotions during challenging situations. This included examining dimensions such as emotional valence, subjective experience of bodily changes and physiological arousal, and whether they perceive their thoughts and emotions as helpful or hindering. Overall, participants tended to view their thoughts as constructive and beneficial in these critical moments. Firefighters represent a highly trained and selective group dedicated to helping others and saving lives, making this conclusion unsurprising. According to Goal Setting Theory (48), a sense of satisfaction naturally arises from achieving significant, challenging, and value-based goals, supported by clear feedback and a strong sense of mastery. Furthermore, it is maybe understandable that firefighters excel at reaching challenging objectives and find satisfaction in their achievements. Emotionally, they tend to perceive their emotions as generally negative in valence but helpful. Indeed, previous research has shown that perception of helpfulness is linked to improved reasoning and an adaptive connexion with the emotion regulation strategy, cognitive reappraisal. According to Karnaze and Levine (19), individuals who view their emotions as beneficial are more likely to employ cognitive reappraisal. This aligns with our findings, as participants reported frequently using cognitive reappraisal as an emotion regulation strategy during stressful operations. Regarding arousal, participants reported high subjective experience of bodily changes and physiological arousal during stressful situations. Nevertheless, previous research suggests that high arousal and negative valence do not necessarily impair task performance when individuals focus on a single task rather than switching between multiple tasks (49). This may explain why the firefighters in our study were still able to perform adaptively despite subjective experiences of negative valence and high arousal.

The correlational analysis also indicates positive relationships between participants' experiences of their thoughts and their emotions during the stressful situations. That is, participants tended to experience their thoughts and emotions as helpful during stressful situations. Notably, subjective reports of bodily arousal increased in proportion to the perceived helpfulness of thoughts and emotions. Despite reporting higher levels of bodily activation (e.g., increased heart rate), participants continued to evaluate their thoughts and emotions as helpful. These correlational findings suggest that, in highly stressful situations, elevated subjective experiences of bodily changes may co-occur with functional cognitive-emotional appraisals among rescue personnel. The results are somewhat consistent with previous research, which demonstrated that cognitive performance (e.g., speed and accuracy) was not impaired among firefighters in stressful operational situations (40). Firefighters also tend to maintain functional emotional states under conditions of intense stress (50).

### 4.2 Emotion regulation as a habitual tendency

The second aim of our study was to examine how firefighters generally regulate their emotions independent of specific operational situations. The results show that firefighters score higher on cognitive reappraisal than on suppression. Thus, the results from the Emotion Regulation Questionnaire (ERQ) were in line with the results from the interview. Cognitive reappraisal is typically associated with greater wellbeing and better social functioning, whereas expressive suppression is linked to higher physiological and cognitive costs (15, 17, 18). In high-stress occupational samples, suppression has repeatedly been identified as a risk factor linking occupational stressors to psychopathology, while reappraisal does not show the same harmful mediating effects (46). Taken together, these findings indicate that firefighters preferentially use cognitive reappraisal as an adaptive emotion regulation strategy, one that supports task performance and resilience under acute stress.

Further, we investigated the relationships among emotion regulation strategies (reappraisal and suppression) and factors such as age and work experience, since these have been shown to correlate with psychological distress among firefighters (12). No significant associations were found between cognitive reappraisal and age, nor between either emotion regulation strategy (cognitive reappraisal or suppression) and work experience. Age-related differences were evident, with suppression decreasing with age. This negative association between age and expressive suppression aligns with previous research showing that older adults tend to rely less on suppression and more on adaptive strategies such as acceptance and reappraisal (51, 52). Nevertheless, when examining the extent to which age could predict variance in suppression, our results indicated only a modest predictive effect (8%). Further studies should draw on larger samples to determine whether similar patterns can be reliably replicated.

### 4.3 Limitations, strengths, and further research

Several limitations warrant consideration. The study's descriptive design limits the ability to draw causal inferences, suggesting that the findings should be interpreted as exploratory rather than explanatory. The CBT-question technique investigated participants' experiences of thoughts and emotions, including perceptions of bodily and physiological changes during specific critical events. The inquiries pertained to past events. Given that memory, particularly episodic memory, is a reconstructive process, the subjective responses to these questions may have been influenced by various biases and interferences [e.g., (53)]. In retrospect, questions about physiological arousal, as well as the usefulness of both thoughts and emotions, could have been influenced by the outcome of the situation, e.g., if it was successful, one could have overestimated the usefulness of both thoughts and emotional or bodily reactions. Additionally, the possible effect of social desirability should be noted. Given the closed professional environment and masculine occupational culture within the fire service, participants may have provided socially approved responses rather than fully reflecting subjective experiences. In general, the interview data must be considered in light of how participants, in hindsight, experienced the situation and what it elicited in terms of both cognitions and emotions. To gain more precise knowledge of firefighters' physiological responses during critical events, future studies must record objective physiological data, such as heart rate variability and cortisol responses. Another important limitation of the study is its small sample size, which may have reduced statistical power. Although the analyses identified several significant associations, small to moderate effects may have remained undetected. The results should therefore be interpreted with some caution, and future studies with a larger sample will be able to provide more precise estimates. In addition, although the regression analysis shows that age explains part of the variation in suppression, the cross-sectional design does not provide a basis for causal interpretations. The result should therefore be understood as a statistical prediction rather than an indication of causal direction.

To preserve participants' own descriptions, emotions such as “stress,” “surprise,” “joy,” and “disappointment” were classified as “mixed emotions,” since several participants reported experiencing multiple emotions in an occupational stressful situation. Although this may be perceived as a methodological vulnerability, it makes sense from an appraisal -theoretical perspective. Mixed emotions are expected in situations where multiple valued goals are simultaneously activated and only partially satisfied (54). In complex professional contexts, such as fire and rescue operations, successful outcomes may co-occur with unavoidable losses, giving rise to combinations of emotions such as relief, sadness, and regret. Our study aligns with empirical research indicating the co-occurrence of mixed emotions in meaning-rich situations (58).

Despite its limitations, a notable strength of this study is its recruitment from a relatively homogeneous cultural and organisational context, which aligns with our aim to examine cognitive and emotional processes within such a setting. Further, the study introduces a novel and promising clinical methodology to firefighter research, CBT. For the first time, CBT has been applied in an operational setting with active-duty firefighters, marking a significant step forward in understanding emotional and cognitive processes under acute stress. One important strength of the CBT question technique is that it identifies both cognitions and emotions (26), and it provides a robust framework for exploring how cognitive appraisals and emotional responses impact problem-solving and emotion regulation in high-pressure occupational environments. By exploring relationships among thoughts, emotions, problem-solving strategies, and emotion regulation during critical situations, this approach offers valuable insights into cognitive patterns that either facilitate or impede effective action. Hansen et al. (9) emphasised the need for a deeper understanding of firefighters' cognitive and emotional processes underlying handling of critical operations, specifically their cognitive appraisals, emotional reactions, and emotion regulation strategies, as well as their beliefs about emotional responses to unforeseen events. The present study addressed this gap by exploring how firefighters experience and navigate these challenges in real-world scenarios. Our integrated perspective connects cognitive-emotional processes with operational outcomes, and it provides a substantive contribution to both research and practise in high-stress professions.

By gaining insight into how firefighters appraise stressful situations, respond emotionally, solve complex operations, and regulate their emotions during high-stress operations—as well as by examining the emotion regulation strategies they commonly employ—the current study presents a more holistic understanding of the cognitive-emotional processes that underpin firefighters' operational performance. Future studies should include larger samples to obtain more reliable statistical results and to investigate further how emotion regulation develops with age.

## Data Availability

The raw data supporting the conclusions of this article will be made available by the authors, without undue reservation.
